# Does affective touch influence the virtual reality full body illusion?

**DOI:** 10.1007/s00221-017-4912-9

**Published:** 2017-03-13

**Authors:** Jutta R. de Jong, Anouk Keizer, Manja M. Engel, H. Chris Dijkerman

**Affiliations:** Experimental Psychology/Helmholtz Institute, Utrecht University, Utrecht, The Netherlands

**Keywords:** Affective touch, Body ownership, Visuo-tactile, Virtual reality, Touch, Full body illusion

## Abstract

The sense of how we experience our physical body as our own represents a fundamental component of human self-awareness. Body ownership can be studied with bodily illusions which are generated by inducing a visuo-tactile conflict where individuals experience illusionary ownership over a fake body or body part, such as a rubber hand. Previous studies showed that different types of touch modulate the strength of experienced ownership over a rubber hand. Specifically, participants experienced more ownership after the rubber hand illusion was induced through affective touch vs non-affective touch. It is, however, unclear whether this effect would also occur for an entire fake body. The aim of this study was, therefore, to investigate whether affective touch modulates the strength of ownership in a virtual reality full body illusion. To elicit this illusion, we used slow (3 cm/s; affective touch) and fast (30 cm/s; non-affective touch) stroking velocities on the participants’ abdomen. Both stroking velocities were performed either synchronous or asynchronous (control condition), while participants viewed a virtual body from a first-person-perspective. In our first study, we found that participants experienced more subjective ownership over a virtual body in the affective touch condition, compared to the non-affective touch condition. In our second study, we found higher levels of subjective ownership for synchronous stimulation, compared to asynchronous, for both touch conditions, but failed to replicate the findings from study 1 that show a difference between affective and non-affective touch. We, therefore, cannot conclude unequivocally that affective touch enhances the full-body illusion. Future research is required to study the effects of affective touch on body ownership.

## Introduction

The way we experience our body as our own and as part of our psychological self is a relatively stable and fundamental component of human daily functioning. Nevertheless, previous literature shows that this sense of self is malleable and prone to manipulation. This was first demonstrated in pioneering research by Botvinick and Cohen (1998) who reported that in the rubber hand illusion (RHI) paradigm participants can feel ownership over a fake rubber hand and experience touch on a rubber hand as if it was applied to their own hand. Recently, virtual reality (VR) techniques emerged and illustrated that using a virtual reality full body illusion (VRFBI), the experience of ownership can even be induced over an entire fake body (Slater et al. 2009, 2010), similar to RHI studies (Botvinick and Cohen 1998). It was found that when participants experienced ownership over a virtual body, they showed physiological responses when the virtual body was being threatened, indicating embodiment over this fake body (Slater et al. 2009, 2010; Petkova et al. 2011a, b). The experience of ownership relies on a combination of both visual and tactile information. Seeing a fake or virtual body (part) being stroked, while feeling synchronous stroking at the corresponding location on the own body that is hidden from view, creates the illusion of ownership over the fake or virtual body (part). This illusory ownership is less prominent or even absent following asynchronous stroking, where the felt stroking does not correspond with the seen stroking (Armel and Ramachandran 2003; Ehrsson et al. 2004; Crucianelli et al. 2013; Botvinick and Cohen 1998). Thus, a key factor of experiencing ownership over a fake or virtual body (part) appears to lie in the generation of a synchronous visuo-tactile (spatial) conflict, as illustrated in both the RHI and VRFBI. Since tactile information is crucial in creating synchronous visuo-tactile conflict it is important to study the contribution of touch to bodily illusions, as we can distinguish between several forms of touch. For example, affective touch informs us about the pleasantness of the touch whereas regular non-affective touch provides information about for example touch discrimination and localization (McGlone et al. 2007; Kaiser et al. 2016; Gordon et al. 2013). Previous literature indicated that affective touch is processed differently from non-affective touch at both anatomical and functional levels. Affective touch has been associated with slow-conducting unmyelinated tactile fibers (CT fibers) that respond optimal to slow stroking velocities of 1–10 cm/s when applied on the hairy skin (McGlone et al. 2007; Löken et al. 2009; Morrison 2012; Björnsdotter and Olausson 2011). The CT fibers that code for affective touch differ from the fast-conducting myelinated tactile fibers which code for non-affective touch (Olausson et al. 2010; Löken et al. 2009; Gordon et al. 2013; Björnsdotter et al. 2010). CT fibers are thought to project to the posterior insula (Gordon et al. 2013; Olausson et al. 2002; Björnsdotter and Olausson 2011), an area responsible for self-awareness (Craig 2009, 2010), emotional experiences (Menon and Uddin 2010; Singer et al. 2009) and encoding of bodily signals (Damasio 2003; Singer et al. 2009). In addition, the insula is considered to contribute to the generation of body ownership (Tsakiris et al. 2007, 2011; Karnath and Baier 2010). Furthermore, areas connected to the insula such as ventral premotor and parietal areas are associated with visuo-tactile integration, which is thought to be the underlying mechanism responsible for creating the experience of ownership over a fake limb (Petkova et al. 2011a, b; Blanke 2012; Ehrsson et al. 2004, 2005; Gentile et al. 2015; Makin et al. 2008; Guterstam et al. 2013).

The link between affective touch and brain areas responsible for visuo-tactile integration and body ownership becomes quite relevant when considering the previously discussed body ownership illusions. Recent studies demonstrated that the strength of illusionary ownership was dependent upon the type of tactile stimulation. It was found that affective touch produced a higher level of ownership over a rubber hand, compared to non-affective touch (van Stralen et al. 2014; Crucianelli et al. 2013; Lloyd et al. 2013). These effects were found when administering synchronous visuo-tactile conflict, and were absent in both conditions when using asynchronous visuo-tactile conflict. These findings, however, are limited to ownership over the hand and it is unclear whether affective touch also modulates a full body illusion. As CT fibers are located in all hairy skin, it can be hypothesized that also in a full body illusion, participants will experience more ownership when inducing the illusion using affective compared to non-affective touch. In two experiments we investigated whether the use of affective touch applied to the abdomen, which also contains c-tactile afferents, when inducing synchronous visuo-tactile conflict, creates a higher level of illusionary ownership over a fake body on a subjective level, compared to non-affective touch. In study 1 we induced the VRFBI using synchronous visuo-tactile stimulation, while in study 2 it was induced using synchronous as well as asynchronous stimulation. For study 1, we expected a difference between affective and non-affective touch, where affective touch would be rated with higher levels of body ownership, compared to non-affective touch. For study 2, we added asynchronous visuo-tactile condition as a control condition. First, we expected the illusion to occur stronger in the synchronous compared to the asynchronous conditions, independent of type of stimulation (affective vs non-affective). Second, similar to study 1 we expected participants to subjectively rate the experience of illusory ownership over the virtual body as higher in the affective compared to the non-affective conditions after synchronous touch, and did not expect differences in ratings between the affective and non-affective condition after asynchronous stroking. The experimental outcomes will provide us with additional knowledge about the apparent plasticity of body representation.

## Study 1

### Methods

#### Participants

Twenty healthy female students participated with an age range between 19 and 38 (M 24.10, SD 3.96). Body Mass Index (BMI) of the participants was assessed using self-reported height and weight, and ranged between 18 and 30.5 (M 22.72, SD 3.00). One participant was excluded due to the experience of discomfort with the experimental procedures. After written informed consent was obtained, participants were verbally instructed by the experimenter. All participants had normal or corrected-to-normal visual acuity and no physical condition that prevented participating in this experiment. Participants were eligible to take part in this study if they were female and at least 18 years of age and was rewarded with course credit. To rule out any gender biases, we only included female who were tested by a female experimenter. We do not expect males to experience the illusion differently since Slater et al. (2010) showed that both female and males experienced ownership over a virtual female body. All procedures were approved by the local ethical committee of Utrecht University.

#### Procedure

The VRFBI was induced twice for each participant, once for the affective touch condition and once for the non-affective touch condition. We compared two conditions: affective touch and non-affective touch: affective touch was characterized by slow stroking velocities of 3 cm/s, whereas non-affective touch was characterized by fast stroking velocities of 30 cm/s. In this study, virtual stroking mimicked actual stroking conducted by the experimenter and participants felt stroking on their own abdomen while seeing the virtual body being stroked in a synchronized way. After each condition, participants were requested to take off the VR goggles, sit down, and complete two questionnaires.

#### Methods and materials

**Fig. 1:**
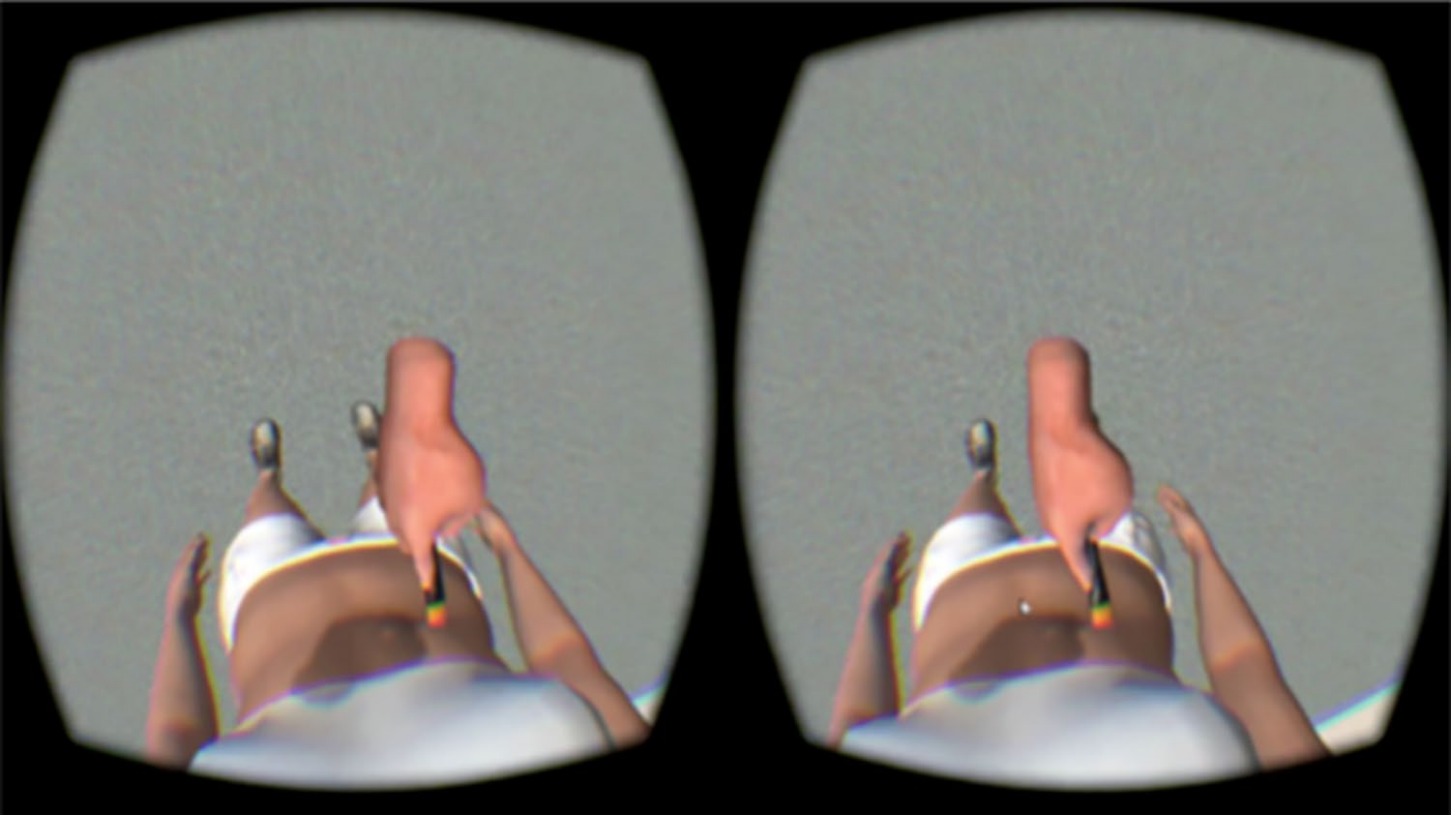
Experimental set-up of the VRFBI

To elicit the VRFBI, participants were instructed to uncover their abdomen and wear VR goggles (Oculus Rift DK2), which prevented them from seeing their own body and their surroundings. In addition, participants were instructed to stand still during the experiment. We used a virtual belly illusion containing a virtual body which was standard for all participants. The body had a waist circumference of 73.94 cm and was standing upright in an empty room. The virtual room was similar to the room where the experiment was conducted. During the procedure, participants were instructed to look down towards their abdomen and saw a static virtual body from a first-person perspective (Fig. 1), see also Serino et al. (2015). Through the VR goggles, participants saw the abdomen of the virtual body being stroked towards the belly button in downward movements by a virtual hand which was holding a brush. The movement of the brush was controlled by the experimenter who stroked the participant’s abdomen with a movement controller (Razer Hydra) to which a soft brush was attached. The total duration of material-skin contact was 90 s per condition. For all conditions, the amount of strokes was controlled for, where affective touch contained three strokes of 1 cm and non-affective touch three strokes of 10 cm.

#### Perceived pleasantness questionnaire

The Experienced Pleasantness of Touch Questionnaire (EPTQ; adapted from Guest et al. 2011) contained seven items which represented either positive (item 1–5) or negative (item 6–7) experience of touch (Table 1). Participants were instructed to rate their subjective experience of each item on a Visual Analogue Scale (VAS), by putting a vertical line on a 10 cm horizontal line, ranging from 0 (Not at all) to 10 (Very much).

**Table 1:** Items of the Experienced Pleasantness of Touch Questionnaire (EPTQ), VAS from 1 to 10, (dis)agreement Adapted version (Guest et al. 2011)

Positive affect	Negative affect
Comfortable	Irritating
Relaxing	Discomfort
Calming	
Soothing	
Enjoyable	

#### Embodiment questionnaire

The Embodiment Questionnaire (EQ; adapted from Piryankova et al. 2014) contained 12 items which evaluated how participants experienced the VRFBI on a subjective level (Table 2). Participants were instructed to rate each statement on a ten-point Likert scale, ranging from 1 (strongly disagree) to 10 (strongly agree). The EQ contained three subscales which measured the amount of ownership, location and agency. Items 1–6 of the EQ, represented the amount of ownership experienced over the virtual body (e.g. I had the feeling that I was looking at myself). The experienced change in location of the body (e.g. It seemed as if I was standing in the same location as the virtual body) was measured by item 7–10 of the EQ. Last, items 11–12 represented the amount of experienced agency over the virtual body (e.g. It seemed as if I had control over the virtual body).

**Table 2:** Embodiment Questionnaire (EQ), Likert scale from 1 to 7 (dis)agreement Adapted from Piryankova et al. (2014)

Question	Subscale
1. Sometimes I felt as if the virtual body was my body	Ownership
2. Sometimes I had the feeling that I was looking at myself	Ownership
3. Sometimes it felt as if I had more than one body	Ownership
4. Sometimes I felt myself somehow connected to the virtual body	Ownership
5. Sometimes it felt like my physical body was changing to take the shape of the virtual body	Ownership
6. Sometimes I had the feeling that the virtual body and I were the same	Ownership
7. Sometimes I had the sensation as if I was feeling the touch at the location at which the virtual abdomen was stroked	Location
8. Sometimes it felt as if the touch I was feeling was located somewhere between my physical body and the virtual body	Location
9. Sometimes I had the sensation as though the touch I felt was caused by the hand with the brush touching the virtual abdomen	Location
10. Sometimes I had the feeling that I was standing in the same location as the virtual body	Location
11. Sometimes I felt I could move the virtual body, if I wanted to	Agency
12. Sometimes I had the feeling that I had control over the virtual body	Agency

### Results

To determine whether the assumption of normality was met, the distribution of the data was examined using the Shapiro–Wilk test of normality. Results suggested that the data was normally distributed (lowest value of *S–W* = 0.92, *p* = .114). There was no correlation (Bonferroni corrected to *α* = 0.007) between BMI and the subscales of the EQ in the affective touch condition (ownership *r* = .23, *p* = .329; location *r* = .16, *p* = .509; agency *r* = .12, *p* = .609) and non-affective touch condition (ownership *r* = −.02, *p* = .928; location *r* = −.13, *p* = .634; agency *r* = .10, *p* = .684). Furthermore, there was no correlation (Bonferroni corrected to *α* =0.007) between age and the subscales of the EQ in the affective touch condition (ownership *r* = −.37, *p* = .104; location *r* = −.20, *p* = .388; agency *r* = −.11, *p* = .644) and non-affective touch condition (ownership *r* = −.25, *p* = .281; location *r* = −.26, *p* = .271; agency *r* = .03, *p* = .894).

#### Pleasantness experience of the VRFBI

**Fig. 2:**
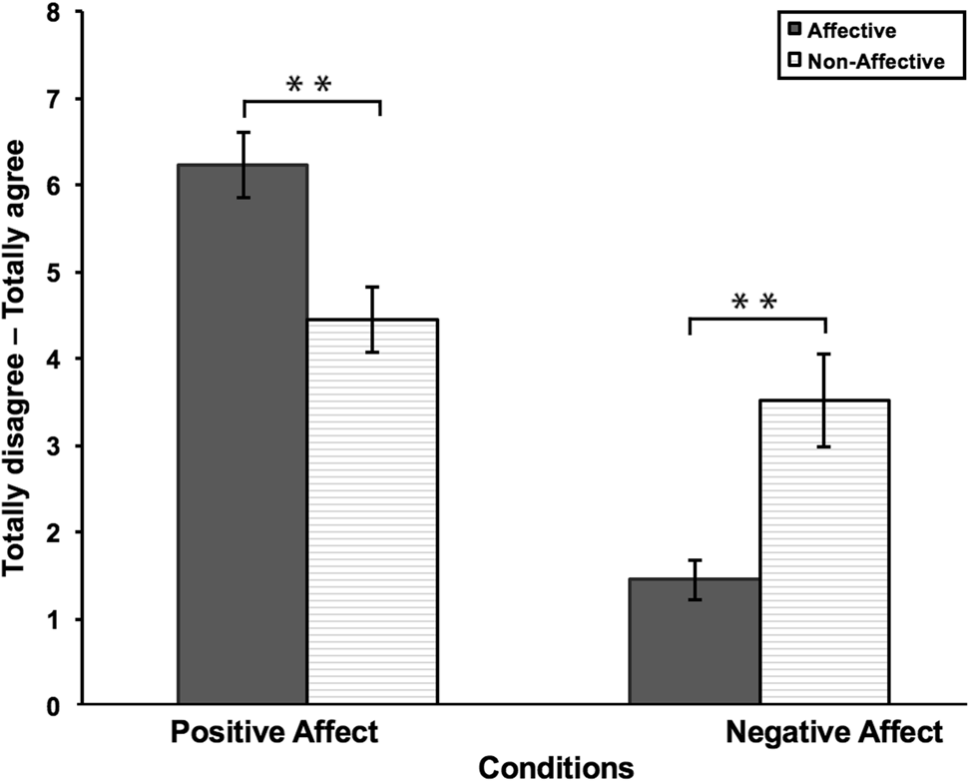
Results of the EPTQ scores for study 1. Affective touch was rated as more positive, compared to non-affective touch. Non-affective touch was rated as more negative, compared to affective touch. *Error bars* represent standard error of the mean. **p < .05

To determine whether the different touch conditions (affective vs non-affective) indeed elicited different ratings of pleasantness we compared the EPTQ scores. Two repeated measures ANOVAs were conducted to compare the amount of perceived pleasantness between the affective and non-affection condition on positive and negative affect items (Table 3; Fig. 2). There was a significant difference in perceived pleasantness of touch between conditions, *F*(1,19) = 22.82, *p* < .000, with affective touch being rated as more positive than non-affective touch. Additionally, there was a significant difference in perceived unpleasantness of touch between conditions, *F*(1,19) = 18.51, *p* < .000, with non-affective touch being rated as more unpleasant than affective touch. Thus, affective touch was rated as more pleasant compared to non-affective touch, whereas the non-affective touch was rated as more unpleasant compared to affective touch.

**Table 3:** Results of the EPTQ scores for study 1

	Affective touch		Non-affective touch	
	M	SD	M	SD
Positive	6.21	1.70	4.43	1.68
Negative	1.44	1.03	3.50	2.38

#### Subjective ownership strength of VRFBI

**Fig. 3:**
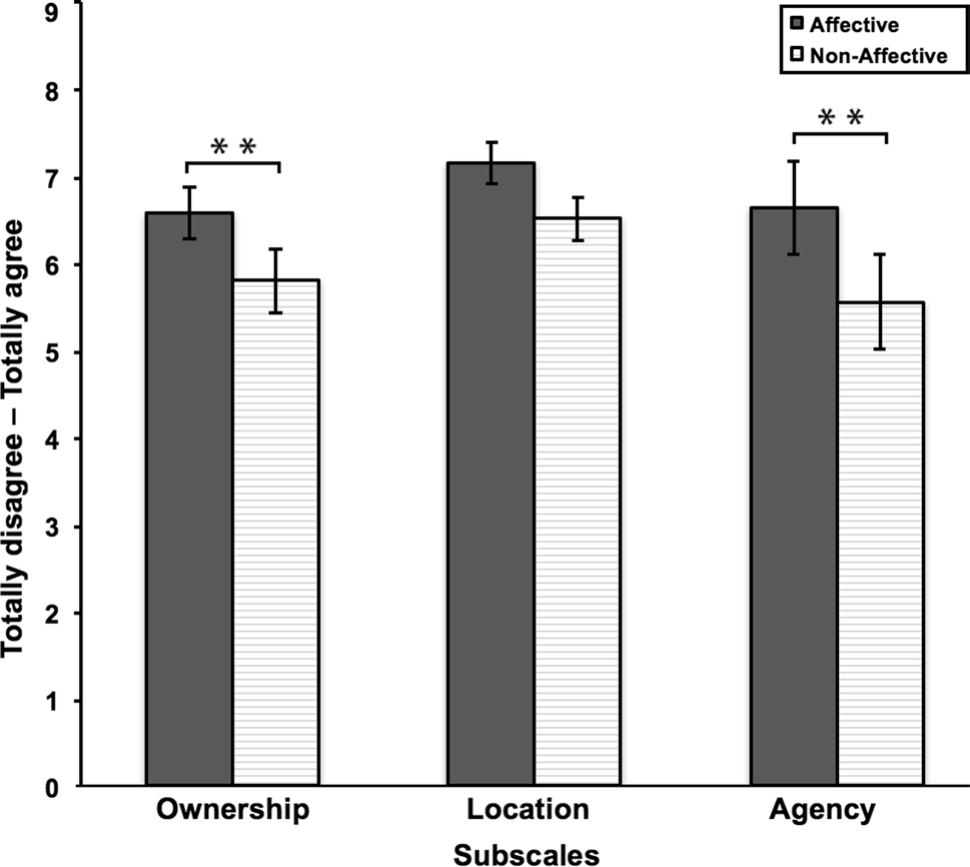
Results of the EQ scores for study 1. Higher ratings were given with regard to ownership and agency for affective touch, compared to non-affective touch. Experienced location did not differ between affective and non-affective touch. *Error bars* represent standard error of the mean. **p < .05

**Fig. 4:**
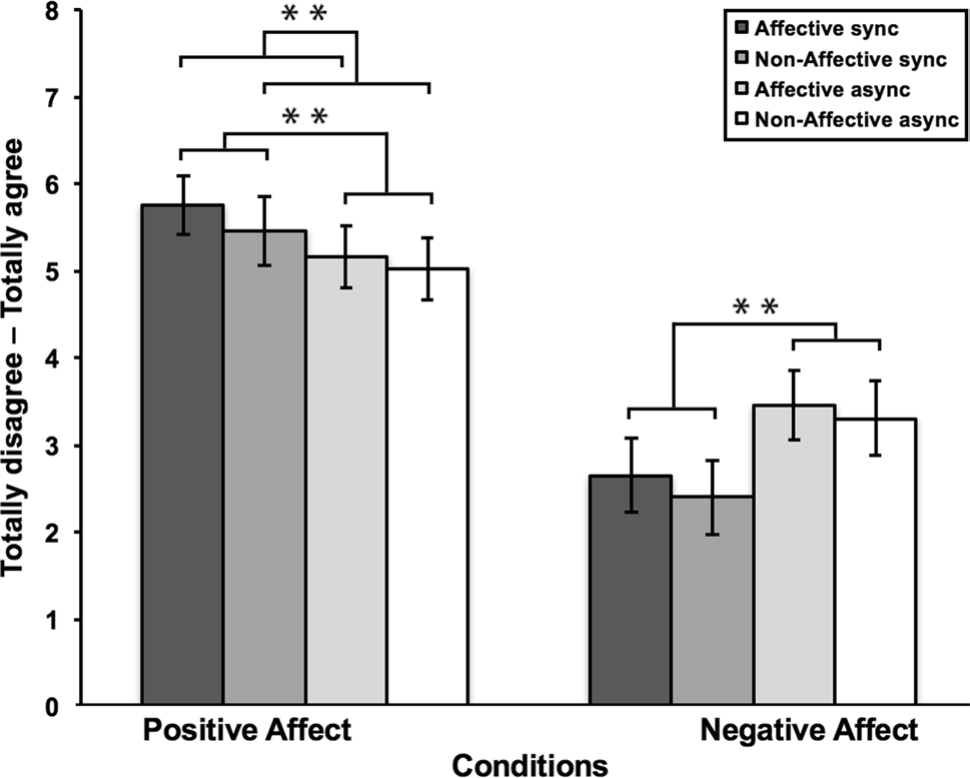
Results of the EPTQ scores for study 2. Synchronous stroking was rated as more pleasant, compared to asynchronous stroking. Affective stroking was rated as more pleasant, compared to non-affective stroking. Affective touch was rated as more pleasant, compared to non-affective touch. For unpleasantness, no difference was found between either synchronous and asynchronous stroking, nor between affective and non-affective stroking. *Error bars* represent standard error of the mean. **p < .05

**Fig. 5:**
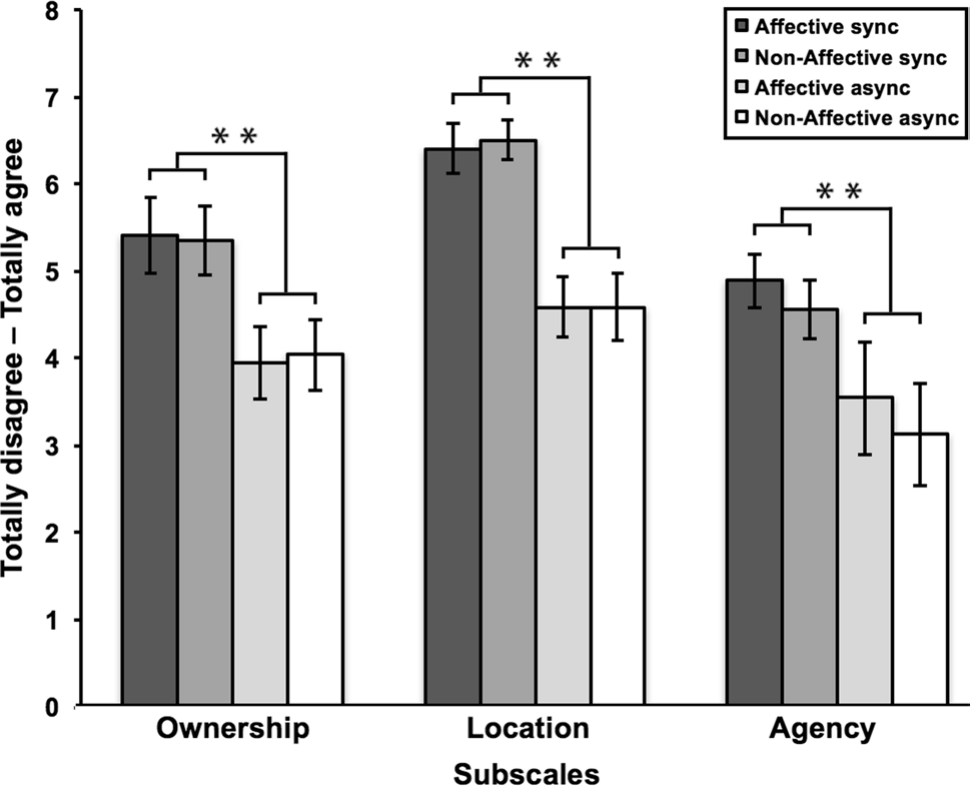
Results of the EQ scores for study 2. Higher ratings were given with regard to ownership, location and agency for synchronous stroking, compared to asynchronous stroking. There was no difference on all scales between affective and non-affective stroking. *Error bars* represent standard error of the mean. **p < .05

Three repeated measures ANOVAs were conducted to compare the amount of perceived ownership, location and agency in the affective and non-affective touch condition (Table 4; Fig. 3). There was a significant difference in body ownership, *F*(1,19) = 16.48, *p* = .001, with affective touch resulting in higher levels of body ownership than non-affective touch. There was no significant difference in location presence, *F*(1,19) = 3.02, *p* = .098, with affective touch not differing in experienced presence at the virtual location, compared non-affective touch. Last, there was a significant difference in agency, *F*(1,19) = 13.25, *p* = .002, with affective touch resulting in higher levels of agency over the virtual body than non-affective touch. Thus, the affective touch condition gave rise to a stronger VRFBI illusion, showing from higher scores on ownership and agency scales. For location, these two conditions did not differ (Figs. 4, 5).

**Table 4:** Results of the EQ scores for study 1

	Affective touch		Non-affective touch	
	M	SD	M	SD
Ownership	6.56	1.35	5.79	1.63
Location	7.13	1.05	6.50	1.10
Agency	6.62	2.38	5.55	2.47

## Study 2

### Methods

#### Participants

Nineteen healthy female students participated with an age range between 19 and 31 (M 22.84, SD 3.37). BMI of the participants was assessed using self-reported height and weight, and ranged between 19 and 25 (M 22, SD 1.40). After written informed consent was obtained, participants were verbally instructed by the experimenter. All participants had normal or corrected-to-normal visual acuity and no physical condition that prevented participating in this experiment. Participants were eligible to take part in this study if they were female and at least 18 years of age and was rewarded with course credit. All procedures were approved by the local ethical committee of Utrecht University. Similar to study 1, we only included female participants who were tested by a female experimenter.

#### Procedures

We induced the VRFBI four times in total, for each participant, which allowed for comparison between induction of the illusion using synchronous affective touch, asynchronous affective touch, synchronous non-affective touch, and asynchronous non-affective touch. Procedures for the synchronous stroking condition were identical to those of Experiment 1. In the asynchronous condition, the felt strokes on the participant’s abdomen did not match the perceived strokes in VR (see also Serino et al. 2015). In this case, the felt strokes alternated with the visually perceived strokes. Asynchronous stimulation was added as a control condition. All conditions were counterbalanced over participants. After each condition, participants were requested to take off the VR goggles, sit down, and complete the EPTQ and EQ questionnaires. Further methods and materials were identical to those of Experiment 1.

### Results

To determine whether the assumption of normality was met, the distribution of the data was examined using the Shapiro–Wilk test of normality. Results suggested that data of the ownership affective synchronous and agency non-affective asynchronous condition were not normally distributed (value of *S–W* = 0.85, *p* = .009, value of *S–W* = 0.89, *p* = .046,). Both skewness and kurtosis were within −2 and + 2, which is considered acceptable to prove a normal distribution (George and Mallery 2010). This, in combination with the robustness of the repeated measures ANOVA we intended to perform, led us to treat all data as normally distributed. There was no correlation (Bonferroni corrected to *α* = 0.003) between BMI and the subscales of the EQ in the affective touch synchronous condition (ownership *r* = −.20, *p* = .408; location *r* = −.08, *p* = .717; agency *r* = −.01, *p* = .943) and non-affective touch synchronous condition (ownership *r* = −.32, *p* = .170; location *r* = −.18, *p* = .474; agency *r* = .08, *p* = .741). Additionally, there was no correlation (Bonferroni corrected to *α* = 0.003) between BMI and the subscales of the EQ in the affective touch asynchronous condition (ownership *r* = −.10, *p* = .674; location *r* = −.17, *p* = .586; agency *r* = .13, *p* = .586) and non-affective touch asynchronous condition (ownership *r* = −.26, *p* = .273; location *r* = −.06, *p* = .778; agency *r* = .07, *p* = .752). There was no correlation (Bonferroni corrected to *α* = 0.003) between age and the subscales of the EQ in the affective touch synchronous condition (ownership *r* = −.07, *p* = .758; location *r* = .09, *p* = .970; agency *r* = −.05, *p* = .826) and non-affective touch synchronous condition (ownership *r* = .02, *p* = .936; location *r* = .08, *p* = .731; agency *r* = −.09, *p* = .700). Additionally, there was no correlation (Bonferroni corrected to *α* = 0.003) between age and the subscales of the EQ in the affective touch asynchronous condition (ownership *r* = .21, *p* = .380; location *r* = .40, *p* = .090; agency *r* = .15, *p* = .537) and non-affective touch asynchronous condition (ownership *r* = −.07, *p* = .765; location *r* = .53, *p* = .019; agency *r* = .38, *p* = .107).

#### Pleasantness experience and subjective ownership of the VRFBI

To determine whether the different touch conditions indeed elicited different ratings of pleasantness we compared the EPTQ scores. We conducted three 2 (synchronicity: synchronous vs asynchronous stroking) × 2 (touch: affective vs non-affective touch) repeated measures ANOVAs, which allowed us to compare the amount of perceived pleasantness between both affective and non-affective touch, for both synchronous and asynchronous conditions, on positive and negative affect items (Table 5). For the positive subscale the results showed a main effect of synchronicity *F*(1,18) = 8.95, *p* = .008, which indicates that participants rated the synchronous conditions are more pleasant, compared to the asynchronous conditions. For stroking, a main effect was found, *F*(1,18) = 42.86, *p* = .000, indicating that affective stroking was rated as more pleasant, compared to non-affective stroking. Furthermore, there was an interaction effect between synchronicity and stroking, *F*(1,18) = 38.68, *p* = .000, indicating that affective touch was rated as more pleasant compared to non-affective touch. The results for the negative subscale showed a main effect of synchronicity *F*(1,18) = 8.87, *p* = .008, which indicates that participants rated the asynchronous conditions as more unpleasant, compared to synchronous stroking. For stroking, no main effect was found, *F*(1,18) = 0.19, *p* = .662, indicating that there was no difference between affective and non-affective stroking on unpleasantness. Furthermore, there was no interaction effect between synchronicity and stroking, *F*(1,18) = 0.11, *p* = .743, indicating that affective touch was rated equally unpleasant as non-affective touch. In sum, these results show that participants rated the experience as more pleasant after the illusion was elicited with affective synchronous touch, compared to non-affective synchronous and asynchronous touch. For the negative ratings, asynchronous stroking was rated more unpleasant compared to synchronous stroking. There were no differences on unpleasantness between affective and non-affective touch.

**Table 5:** Results of the EPTQ scores for study 2

	Affective sync		Affective async		Non-affective sync		Non-affective async	
	M	SD	M	SD	M	SD	M	SD
Positive	5.72	1.47	5.12	1.53	5.42	1.66	4.99	1.55
Negative	2.63	1.81	3.44	1.71	2.51	1.78	3.28	1.86

**Table 6:** Results of the EQ scores for study 2

	Affective sync		Affective async		Non-affective sync		Non-affective async	
	M	SD	M	SD	M	SD	M	SD
Ownership	5.41	1.86	3.94	1.83	5.35	1.72	4.04	1.80
Location	6.51	1.11	4.73	1.40	6.51	0.93	4.63	1.71
Agency	5.05	2.18	3.63	2.11	4.55	2.33	3.25	1.78

#### Subjective ownership strength of VRFBI

We conducted three 2 (synchronicity: synchronous vs asynchronous stroking) × 2 (touch: affective vs non-affective touch) mixed repeated measures ANOVA’s, which allowed us to compare for each EQ subscale the four conditions in which the VRFBI was induced. For the ownership subscale the results showed a main effect of synchronicity *F*(1,18) = 18.60, *p* < .001, indicating that participants rated EQ ownership statements as higher after synchronous stroking, compared to asynchronous stroking. No main effect for stroking was found, *F*(1,18) = 0.02, *p* = .896, indicating that both affective and non-affective touch elicited an equally strong subjective experience of the illusion in terms of ownership over the avatar. There was no interaction between synchronicity and stroking, *F*(1,18) = 0.54, *p* = .474. Similar results were found for the location subscale. We identified a main effect of synchronicity, *F*(1,18) = 20.76, *p* < .001, indicating a stronger experience of change in location after synchronous compared to asynchronous induction of the FBI. No main effect of touch was found, *F*(1,17) = 0.18), *p* = .680, indicating that location statements of the EQ were similar after affective and non-affective touch. No interaction between synchronicity and stroking was observed, *F*(1,17) = 0.08, *p* = .781. For the agency subscale similar effects were found as for the other two subscales. There was a significant main effect of synchronicity, *F*(1,17) = 10.79, *p* = .004, indicating that participants rated EQ agency statements as higher after synchronous compared to asynchronous induction of the illusion. No main effect for touch was found, *F*(1,17) = 3.70, *p* = .071, indicating that participants experienced an equal strong sense of agency over the avatar after affective and non-affective touch. No interaction between synchronicity and stroking was found, *F*(1,17) = 0.07, *p* = .793. Taken together the results show that participants experienced the illusion as stronger with regard to ownership, location, and agency when it was induced using synchronous compared to asynchronous stroking. Type of touch (affective or non-affective) did not affect the subjective experience of the illusion in this study with respect to experienced ownership, location, agency (Table 6).

## Discussion

In the current study we examined the influence of affective touch on body ownership, measured through a VRFBI.

Following previous work on the RHI (van Stralen et al. 2014), we expected that the use of affective touch applied to the abdomen would elicit a stronger full body illusion, compared to non-affective touch. In addition, we expected that this effect would only be present when the perceived and felt stroking was synchronous, and absent when it was administered asynchronous, as the latter condition serves as a control condition in which the illusion is not elicited.

Two studies were conducted and for both studies we found that affective touch was rated as more pleasant, compared to non-affective touch, from which we concluded that our slow stroking conditions indeed reflected affective touch. Within the first study, we compared affective touch to non-affective touch. The second study compared synchronous and asynchronous affective touch, as well as synchronous and asynchronous non-affective touch. The results from study 1 showed that affective touch elicited a stronger sense of self-reported ownership over a virtual body and a stronger sense of agency over the virtual body, compared to non-affective touch. For study 2, we found differences between synchronous and asynchronous touch, where synchronous stroking was rated higher on all illusion scales, compared to asynchronous, for both affective and non-affective touch. This was in line of our expectations, since asynchronous stroking was used a control condition, similar to previous studies (Slater et al. 2010; Armel and Ramachandran 2003; Ehrsson et al. 2004; Crucianelli et al. 2013; Botvinick and Cohen 1998). In addition, we compared the difference scores between affective and non-affective touch, for both synchronous and asynchronous conditions. We expected to find an interaction between touch and synchronicity based on previous literature and our results from study 1, however, did not find these results for study 2.

When interpreting study 1, higher level of embodiment of the virtual body reported by participants after affective touch is in line with previous findings from RHI studies. Research by Crucianelli et al. (2013) shows some indication of modulated embodiment using affective touch, this was further investigated by van Stralen et al. (2014), who confirmed with their data that affective touch increases ownership over a rubber hand, compared to non-affective touch. Thus, the results from study 1 indicate that the modulatory effects of affective touch found in RHI studies, can be generalized to an entire (virtual) body, instead of only a hand, using a VRFBI. Several possible factors may contribute to the enhanced illusion during slow affective stroking. First, the increased experience of embodiment in the affective touch condition may be related to activation of an additional tactile channel. The use of affective touch activated CT fibers in hairy skin which respond to stroking velocities between 1 and 10 cm/s (McGlone et al. 2007; Löken et al. 2009; Morrison 2012; Björnsdotter and Olausson 2011) and are thought to project to the posterior insula (Gordon et al. 2013; Olausson et al. 2002). This area is associated with the generation of body ownership (Tsakiris et al. 2007, 2011; Karnath and Baier 2010). Second, since several studies suggest that the insula is also involved in processing emotional experiences (Menon and Uddin 2010; Singer et al. 2009), the affective tactile input could have led to increased multisensory integration. Interestingly, a previous study using virtual reality compared the effects on body ownership when merely seeing pleasurable or neutral touch on a virtual hand. This study reported that when comparing viewing pleasurable to neutral touch, there was no significant difference between the amount of reported ownership over the virtual hand (Fusaro et al. 2016). This further indicates that when using affective touch to induce a body ownership illusion, the increased experience of ownership can be attributed to the tactile input only. Thus, for our study the use of affective touch may have resulted in an increased sense of embodiment over the virtual body due to additional tactile input with emotional valence, which is important and relevant for humans.

Following Piryankova et al. (2014), we also assessed location and agency after the VRFBI and found results similar to increased levels of ownership after the affective touch condition. Specifically, the location subscale represented the amount of experienced presence in the virtual location, whereas for RHI studies, location refers to the change in perceived hand location towards the rubber hand, known as proprioceptive drift. Since the virtual body was in the same physical location as the body of the participant, this could explain why participants did not experience a difference virtual location presence between conditions. However, participants reported feeling more agency over the virtual body and had a stronger illusion of being able to move this body when affective touch was applied, compared to non-affective touch. The higher scores on agency for the affective touch condition compared to the more neutral experience of agency in the non-affective touch condition are interesting when considering that both the participant and virtual body did not move during the experiment.

For study 2, we found an overall difference between the synchronous and asynchronous conditions, but did not find a main nor interaction effect for the type of touch, indicating that there were no differences between affective and non-affective touch on body ownership. This is unexpected, because our first study, as well as previous literature (van Stralen et al. 2014; Crucianelli et al. 2013; Lloyd et al. 2013) did find higher levels of ownership for affective touch, compared to non-affective touch, Since the absolute means of both the affective and non-affective touch conditions point in the same direction as found in study 1, we suspect that study 2 was underpowered since this study had more conditions compared to study 1. Furthermore, we suspect that the used setup in this case was not suitable to measure both difference between both two forms of touch and (a)synchronicity. When asking participants after study 2 what they had experienced, most reported being occupied with the differences between the synchronous and asynchronous conditions, since this was most salient during the experiment. The subtler difference between different stroking velocities was less noticeable. When taken all these factors into account, we expect that with more participants, the previous findings from study 1 will be present again.

Another interesting point is related to the subjective levels of experiences ownership over a fake body. van Stralen et al. (2014) found consistent differences between affective and non-affective stroking on the rubber hand illusion only for the proprioceptive drift measure, but not at a subjective level when using questionnaires. In our studies we were unable to use the measure of proprioceptive drift since the physical and virtual body were in the same location. The location subscale stems from a questionnaire used in a study by Piryankova et al. (2014) and here they found differences between synchronous and asynchronous touch for a virtual body from a first-person-perspective. In our second study, we indeed find these differences. However, the location subscale may assess a different aspect of the illusion compared to proprioceptive drift. Thus, a different measurement could be more appropriate to study the effects of affective touch on a full-body illusion. Unfortunately, the current set-up did not allow us to assess proprioceptive drift for the full-body illusion. Assessing proprioceptive drift with a full-body illusion may, however, be possible with when using a third-person perspective full-body illusion. Although studies demonstrated that a first-person perspective aspect causes higher levels of experienced ownership, compared to a third-person perspective (Maselli and Slater 2014; Pavone et al. 2016), it would be interesting to study the differences in affective and non-affective touch with a VRFBI with a third-person perspective.

If affective touch indeed leads to higher levels of ownership, this would be a benefit for future studies that use visuo-tactile stimulation for body ownership illusions. Body ownership problems occur in several neurological conditions due to for example brain lesions (Vallar and Ronchi 2009), limb amputation or dysfunctioning nerve connections (Maselli and Slater 2013). Body ownership illusions are also used to study pain perception. Previous studies found that when a painful stimulus was applied to a participant’s body, embodiment over a virtual body led to an increase in pain threshold (Longo et al. 2009; Hänsell et al. 2011; Romano et al. 2014, 2016; Martini et al. 2014, 2015). We speculate that in this case, higher ownership levels during the illusion may lead to a larger decrease in pain threshold. In this case, findings concerning affective touch would be beneficial for pain management interventions that use embodiment over a virtual body to treat pain. The current study may provide information for future research focusing on the effects of affective touch on body ownership. For example, affective touch may not only be advantageous when dealing with patients with body part disownership (van Stralen et al. 2013), but also in patient groups whose body awareness problems may not be limited to one arm or leg, such as anorexia nervosa (Keizer et al. 2011, 2012, 2013) or schizophrenia (Thakkar et al. 2011).

In conclusion, the findings from our first study are in line with previous RHI studies, affective touch modulates several aspects of subjective embodiment over a virtual body. Our second study demonstrates that when comparing synchronous to asynchronous affective touch, and for non-affective touch, that in the synchronous condition participants experience higher levels of ownership over the virtual body, compared to the asynchronous conditions. Although we were not able to replicate the findings from study 1, we suggest that with a larger sample it may be possible to establish differences between affective and non-affective touch on body ownership. Further research is required to investigate the effects on affective touch on body ownership, as found in both performed studies and previous literature. Findings regarding body ownership manipulations provide more knowledge about the modulatory effects of touch during body ownership illusions and more insight in the experience of embodiment.
